# Boosting Abscopal Response to Radiotherapy with Sargramostim: A Review of Data and Ongoing Studies

**DOI:** 10.7759/cureus.4276

**Published:** 2019-03-19

**Authors:** Robyn Leary, Robert B Gardner, Colleen Mockbee, Debasish F Roychowdhury

**Affiliations:** 1 Oncology, Partner Therapeutics, Lexington, USA

**Keywords:** myeloid growth factor, combination immunotherapy, cancer, immunotherapy, metastatic tumors, immuno-oncology, radiotherapy, pancreatic cancer, non–small cell lung cancer, melanoma

## Abstract

Drug development in oncology today routinely focuses on approaches that utilize the patients’ immune system to destroy the malignancy. Combinatorial approaches of antineoplastic agents, both new and old, are being incorporated in the armamentarium of cancer treatments. The overarching goal of therapy remains the achievement of a complete and durable response with long term remission or cure. One approach in advancing treatment is aimed at strategies that improve immunological memory to induce long lasting immunity against the tumor. Although radiation therapy has not traditionally been thought to elicit an immunological effect, an increasing number of reports document the induction of an immune response against a tumor that kills cancer cells distant to the original site of treatment after local irradiation to a tumor. This phenomenon is called an abscopal effect. Since radiation alone is rarely associated with such a response, it is being combined with immuno-oncology drugs in an attempt to enhance response. One such strategy combines sargramostim, a recombinant human granulocyte macrophage colony stimulating factor (rhu GM-CSF), with radiotherapy. GM-CSF is a cytokine secreted by multiple cells types that promotes maturation of dendritic cells and enables the presentation of tumor-associated antigens to generate a T-cell response. This review article discusses the outcomes of clinical trials and case reports examining the efficacy and safety of combining radiation therapy with this immunomodulatory agent. We will also examine future studies and challenges facing the translation of this therapeutic approach.

## Introduction and background

**Figure 1:**
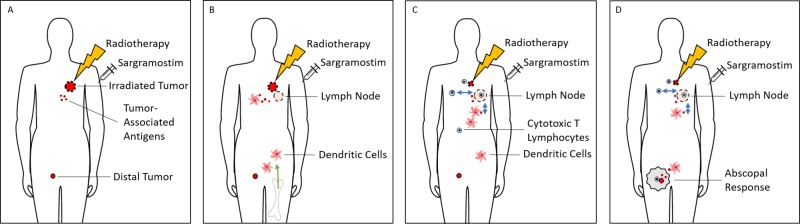
Induction of Abscopal Response by Radiotherapy and Sargramostim A) Radiotherapy with sargramostim is under investigation as a therapy for the treatment of solid tumors. Radiotherapy delivered to a tumor results in the release of tumor-associated antigens and the induction of a proinflammatory signaling cascade. B) Sargramostim supports myelopoiesis (green arrow) and enhances proliferation and differentiation of dendritic cells which process tumor-associated antigens from the irradiated tumor. C) Cross-presentation of tumor-associated antigens to CD8+ T cells by dendritic cells results the activation and migration of cytotoxic T lymphocytes (blue arrows) enhancing the antitumor response. D) The observation of circulating dendritic cells and cytotoxic T cells mounting an attack on distal tumors untreated by radiotherapy demonstrates an abscopal response.

The synergy between immunotherapy and radiotherapy is being used to enhance therapeutic responses. One such strategy combines sargramostim, a recombinant human granulocyte macrophage colony stimulating factor (rhu GM-CSF) with radiotherapy (Figure 1). GM-CSF, a cytokine is involved in the production and maturation of a broad range of hematopoietic cells. The biological functions of GM-CSF are mediated through binding of its receptor (GM-CSFR) which triggers downstream signaling pathways such as Janus kinase-signal transducer and activator of transcription (JAK-STAT), phosphoinositide 3-kinase (PI3K), mitogen-activated protein kinase (MAPK), and nuclear factor-κB (NF-κB) signaling [1-3]. GM-CSF acts at both early and late stages of cellular differentiation and is thus necessary for the production of neutrophils, monocytes/macrophages, eosinophils, and myeloid dendritic cells [4-5]. This potent cytokine also facilitates the maturation and migration of dendritic cells to lymph nodes. Dendritic cells play an important role in the presentation of antigens and priming for primary and secondary T cell response [6]. Considering these effects, sargramostim has been used as a single agent as well as in combination both for antitumor treatment effects and as a vaccine in oncology trials [7]. The overarching goal of these therapeutic approaches combining sargramostim is the potential reversal of the host’s immune tolerance to its own tumor-associated antigens to evoke long-lasting antitumor immunity.

Radiation therapy given locally can induce an immune response against a tumor that kills cancer cells distant to the original site of treatment [8]. This phenomenon is referred to as an abscopal response. In addition to inducing cytotoxic DNA damage, radiation releases immunostimulatory cytokines [9-10]. The release of DAMPs (damage-associated molecular patterns) acts as a danger signal to stimulate dendritic cell activation and antigen presentation inducing immunogenic cell death. Radiation bolsters CD8+ T cell infiltration and natural killer (NK) cell activity in the tumor microenvironment [11-12]. Increasingly, radiation therapy is being combined with immuno-oncology drugs to enhance the effect of these agents [13].

## Review

Clinical trials

Golden and colleagues published the first Phase II study investigating radiotherapy in combination with sargramostim in patients with metastatic solid tumors that included an endpoint to assess abscopal response [14]. Subjects with metastatic cancer qualified for enrollment if they had a minimum of three distinct measurable tumors ≥1 cm. All patients had adequate bone marrow function with an absolute neutrophil count greater than 1500 cells per µL and platelet concentration greater than 50,000 per µL. These patients continued on the same chemotherapy or hormonal therapy they were on prior to their enrollment; however, dosing was adjusted according to radiotherapy in order to minimize toxicities. Patients were not included if they received immunotherapy in the four weeks prior to enrollment. Subjects with brain metastases were eligible for the trial; however, these lesions were not evaluated in the study.

A total of 41 patients with lung, breast, thymic, urothelial, ovarian, eccrine, or cervical cancer and confirmed metastatic cancer with stable or progressing disease were treated with concurrent radiation (35 Gy in ten fractions, over two weeks) and sargramostim (125 μg/m2 daily for two weeks, starting one week after the start of each course of radiotherapy). Lesions were treated sequentially, with the second course of therapy targeting a second distant metastatic site. Computed tomography (CT) scans evaluated patient response 7-8 weeks after the start of treatment.

The primary endpoint was to determine the proportion of patients with an abscopal response, as defined by a reduction of at least 30% in any measurable non-irradiated lesion ≥1 cm. For this endpoint to be achieved, ≥20% of patients needed to exhibit an abscopal response. The secondary endpoints were to evaluate the safety and overall survival associated with abscopal responses. Lymphocytes were monitored by complete blood counts as an exploratory endpoint. The study is complete and registered with ClinicalTrials.gov [NCT02474186]. 

The treatment was well tolerated. No patients discontinued treatment due to the toxicity of the regimen, and no instances of dose reduction of sargramostim or radiation were required. Grade 3 or 4 toxicities primarily started during the first course of therapy, with the most common Grade 3 or 4 adverse events being fatigue (six patients) or hematological (10 patients) in nature.

Overall, the study met its prespecified margin for activity, with more than 20% of patients having an abscopal response. In 27% of patients, abscopal responses were observed and were predictive of improved overall survival (20.98 vs. 8.33 months). When patients were divided based on their type of response, the risk of death for non-responders was more than twice that of responders. Responses were observed in patients with lung cancer (4 of 18 patients) including two complete responses, breast cancer (5 of 14 patients), and thymic cancer (2 of 2 patients). The median follow-up was 5.62 years.

Differences in baseline hematological parameters were observed between subjects with and without an abscopal response, possibly indicating a subset of patients may respond better to this treatment strategy. Although this trial did not incorporate comprehensive profiling of immune cells, hematological parameters included baseline levels of hemoglobin, albumin, and white blood cell count. Abscopal responders presented with a lower baseline neutrophil to lymphocyte ratio than non-responders (2.29 vs 4.24). No significant differences in the mean hemoglobin and albumin concentrations were observed. The possibility that neutrophils or other immunosuppressive constituents of the tumor microenvironment may limit immune responses warrants further investigation in future trials [15-16].

Case reports

Recently, two case reports documenting an abscopal response provide further evidence for combining sargramostim with radiotherapy [7,17]. A patient with metastatic pancreatic cancer treated with gemcitabine and paclitaxel albumin presented with metastases in the liver and right pleura in the presence of rising CA199. Treatment with tyrosine kinase inhibitor, apatinib was intolerable due to gastrointestinal side effects and further targeted therapy was refused [7]. The patient experienced jaundice and palliative radiotherapy given as a total dose of 45 Gy delivered in fifteen fractions for three weeks to alleviate abdominal pain caused by the primary tumor. Subcutaneous injections of sargramostim were given for 14 days, (125 μg/m2) daily for two weeks beginning one week after the initiation of radiotherapy to the end of treatment. Reduction of the primary tumor and metastases was achieved and stable disease documented by CT at one month and three months post therapy, suggesting this therapeutic approach warrants further research for the treatment of pancreatic cancer.

A case report described an abscopal response in a patient diagnosed with stage IIIB, unclassifiable non-small cell lung cancer (NSCLC) treated with local radiotherapy in combination with oncothermia and sargramostim [18]. The subject presented with a 9.5 cm cavitary lesion in right lobe with regional and metastatic lymph nodes. After refusing chemotherapy and requesting an alternative treatment option, radiation with a dose of 1.7 cGy in 28 daily fractions for 5-6 treatments per week was followed by three oncothermia treatments post radiation. Two weeks after treatment, daily subcutaneous sargramostim injections followed for 10 days, (250 μg/m2 daily). This treatment was well tolerated. Multiple metastatic lymph nodes distal from the site of radiation demonstrated nearly a complete remission. Some data suggest this approach could enhance the immune response by locally increasing tumor oxygenation, perfusion, natural killer cell activity and trafficking of dendritic cells to the lymph nodes. Hyperthermia may also assist in overcoming immune tolerance [19-21]. 

Future directions

Further research to understand the predictors of response to therapy and ways to overcome immune tolerance are necessary to improve patient outcomes. One suggestion by Golden et al. is to combine a checkpoint inhibitor with radiotherapy and sargramostim. The proof of concept for combining sargramostim with a checkpoint inhibitor was established in advanced melanoma. In a randomized Phase II trial, assessment of sargramostim in combination with ipilimumab in treating patients with unresectable stage III/IV melanoma showed a prolonged overall survival (HR= 0.64, p=0.01; 17.5 months vs 12.7 months, p=0.01). Patients were treated with ipilimumab (10 mg/kg, intravenously) every three weeks for four doses, then every 12 weeks, and were administered sargramostim 250 μg subcutaneously on the first two weeks of each three-week cycle. Furthermore, the combination of sargramostim and ipilimumab resulted in lower toxicity compared with ipilimumab alone [22]. Like other cytokines, GM-CSF has both immune effector and regulatory functions and both of these effects may have important utilities in patients with cancer as was demonstrated with improved efficacy and reduced toxicity.

To get a better understanding of future studies investigating the combination of radiotherapy in conjunction with sargramostim, the ClinicalTrials.gov database was queried using the search terms: GM-CSF, GMCSF, Granulocyte-Macrophage Colony-Stimulating Factor, sargramostim and Leukine. This search identified 445 trials, which were then individually assessed to determine if the listed trial included radiation as treatment modality in each study (database accessed December 8, 2018). A total of eight ongoing clinical trials aiming to test the combination of radiotherapy in conjunction with GM-CSF were identified (Table 1). In most of the studies, abscopal response is not described as primary or secondary endpoint, and the majority of the studies do not evaluate the combination of radiotherapy and sargramostim with a checkpoint inhibitor.

**Table 1:** Ongoing Clinical Trials Combining Radiation and Sargramostim AE = Adverse Events, D = Day, DLT = Dose Limiting Toxicity, DOR = Duration of Response, GBM = Glioblastoma, IMRT = Intensity Modulated Radiotherapy, NSCLC = Non-Small Cell Lung Cancer, ORR = Objective Response Rate, OS = Overall Survival, PFS = Progression Free Survival, rhGM-CSF = Recombinant Human Granulocyte Macrophage Colony Stimulating Factor, SBRT = Stereotactic Body Radiation Therapy, TTNT = Time to Next Treatment, Wks = Weeks. If sargramostim or Leukine was not designated as a drug on ClinicalTrials.gov, the intervention was referred to as recombinant GM-CSF above.

Trial ID	Title	Phase	Condition	Radiation	Intervention	Primary Endpoints	Secondary Endpoints	Exploratory Endpoints
NCT02383212	Study of REGN2810 (Anti-PD-1) in Patients With Advanced Malignancies	I	Multiple Cancers	Hypofractionated Radiotherapy	Chemotherapy regimens containing Cyclophosphamide, Carboplatin, Paclitaxel, Pemetrexed and/or Docetaxel, Anti-PD-1 (REGN2810) and Sargramostim	AE, Dose Limiting Toxicities	RECIST, irRC	None
NCT02623595	A Study of SBRT in Combination With rhGM-CSF for Stage IV NSCLC Patients Who Failed in Second-line Chemotherapy	II	Lung Cancer	SBRT 50Gy/5F D 1 to D 5, 21 D cycle	Recombinant GM-CSF	Abscopal Effect Rate	OS, Incidence (AE), PFS, ORR, Abscopal Effect Rate, Incidence Treatment-Related AE, Immune Related AE	T Cell Count, Ratio of Effector T Cells: Regulatory T Cells
NCT02663440	Trial of Hypofractionated Intensity Modulated Radiation Therapy With Temozolomide and Granulocyte-Macrophage Colony-Stimulating Factor for Patients With Newly Diagnosed Glioblastoma Multiforme	II	Glioblastoma	Hypofractionated IMRT	Temozolomide and Recombinant GM-CSF	PFS	None	None
NCT02677155	Sequential Intranodal Immunotherapy Combined with Anti-PD1 (Pembrolizumab) in Follicular Lymphoma	II	Lymphoma	8 Gy on D 2 of 5 D cycle	Pembrolizumab, Rituximab and Sargramostim	ORR, Change in Tumor Load	DOR, PFS, TTNT, OS, Change in Tumor Volume, Safety, Antitumor T cell Responses (Blood)	None
NCT02976740	SBRT Combination With rhGM-CSF and Tα1 for Stage IV NSCLC Patients Who Failed in Second-line Chemotherapy	II	Lung Cancer	SBRT 50Gy/4-10F from D 1 to D 10	Thymosin Alpha and Recombinant GM-CSF	Abscopal Effect Rate	OS, Incidence AE, ORR, Incidence Immune-Related AE	None
NCT03113851	Abscopal Effect of Radiation in Combination With rhGM-CSF for Metastatic Non-small Cell Lung Cancer	II	Lung Cancer	3.5 Gy/fraction; total dose of 35 Gy/ 10 fractions over 2 Wks	Recombinant GM-CSF	Abscopal Effect Rate	OS, PFS	None
NCT03392545	Combination of Immunization and Radiotherapy for Recurrent GBM	I	Glioblastoma	Radiotherapy Not Specified	Poly I:C and Recombinant GM-CSF	Incidence Treatment Related AE	OS, PFS	None
NCT03489616	Chemotherapy Combination With Local Radiotherapy and rhGM-CSF for Oligometastatic Stage IV NSCLC Patients	II	Lung Cancer	4Gy per time (or BED ＞45Gy） D 2 to D 15 in cycle of 21 D	Pemetrexed and Recombinant GM-CSF	PFS	Abscopal Effect Rate, OS	None

## Conclusions

These studies and reports suggest the combination of radiation therapy with sargramostim may result in an abscopal response in patients with solid tumors, but further research is necessary to validate these findings. The comparison of overall survival for patients with or without an abscopal response is interesting; however, interpretation is difficult without control arms which were not previously included in the study design. Furthermore, without prospective monitoring, one cannot draw conclusions as to whether antitumor T cell responses are enhanced by this therapeutic approach. It is imperative to collect more information on the immunological and molecular phenotypes of abscopal responders vs non-responders. For example, immunosequencing approaches would provide investigators with a better picture as to what components of the tumor are being recognized. This could also assist in evaluating the extensiveness of the adaptive immune response. As previously described, cytotoxic T lymphocytes kill tumor cells by recognizing tumor-associated antigens presented with major histocompatibility complex (MHC) molecules. To characterize which antigens these T cells recognize, immunosequencing makes it possible to see the diversity of the T cell receptor repertoire. Analysis of T cell clonotypes which infiltrate a given lesion provides insight into the T cell response as well as a repertoire of response. Together, these data could provide us with key mechanistic insights into the potential synergy of GM-CSF and radiation. A carefully designed and executed study is an important next step to determine the potential of this therapeutic approach.
